# Epidemiological Investigation of a Rift Valley Fever Outbreak in Humans and Livestock in Kenya, 2018

**DOI:** 10.4269/ajtmh.20-0387

**Published:** 2020-08-03

**Authors:** Abdala Hassan, Mathew Muturi, Athman Mwatondo, Jack Omolo, Bernard Bett, Solomon Gikundi, Limbaso Konongoi, Victor Ofula, Lyndah Makayotto, Jacqueline Kasiti, Elizabeth Oele, Clayton Onyango, Zeinab Gura, Kariuki Njenga, Peninah Munyua

**Affiliations:** 1 Field Epidemiology and Laboratory Training Program, Ministry of Health, Nairobi, Kenya;; 2 Kenya Zoonotic Disease Unit, Nairobi, Kenya;; 3 International Livestock Research Institute, Nairobi, Kenya;; 4 National Public Health Laboratory Service, Nairobi, Kenya;; 5 Kenya Medical Research Institute, Center for Virus Research, Nairobi, Kenya;; 6 Division of Disease Surveillance and Response, Ministry of Health, Nairobi, Kenya;; 7 Directorate of Veterinary Services, Central Veterinary Laboratory, Nairobi, Kenya;; 8 Division of Global Health Protection, Centers for Disease Control and Prevention, Nairobi, Kenya;; 9 Washington State University Global Health Program-Kenya, Washington State University, Pullman, Washington

## Abstract

On the last week of May of 2018, a community-based syndromic surveillance system detected mass abortions and deaths of young livestock in northeastern Kenya. Two weeks later, Rift Valley fever (RVF) was confirmed in humans presenting with febrile illness and hemorrhagic syndrome in the same region. A joint animal and human response team carried out an investigation to characterize the outbreak and identify drivers of disease transmission. Here, we describe the outbreak investigation and findings. A total of 106 human cases were identified in the months of May and June 2018: 92% (98) and 8% (8) of these cases occurring in the northern and western regions of Kenya, respectively. Seventy-six (72%) were probable cases, and 30 (28%) were laboratory confirmed by ELISA and/or PCR. Among the confirmed cases, the median age was 27.5 years (interquartile range = 20), and 60% (18) were males. Overall, the case fatality rate was 7% (*n* = 8). The majority of the confirmed cases, 19 (63%), reported contact with livestock during slaughter and consumption of meat from sick animals. All confirmed cases had fever, 40% (12) presented with hemorrhagic syndrome, and 23% (7) presented with jaundice. Forty-three livestock herds with at least one suspect and/or confirmed animal case were identified. Death of young animals was reported in 93% (40) and abortions in 84% (36) of livestock herds. The outbreak is indicative of the emergence potential of RVF in traditionally high- and low-risk areas and the risk posed by zoonosis to livestock keepers.

## INTRODUCTION

Rift Valley fever (RVF) is a vector-borne zoonosis caused by *Phlebovirus* in the family Phenuiviridae. Globally, epidemics of RVF are most frequent in the horn of Africa’s Rift Valley region and the Arabian Peninsula.^1^ Outbreaks, however, have been reported in islands off the East African coast: Madagascar and French Island of Mayotte; parts of West, South, and North Africa; and in the Arabian Peninsula countries of Saudi Arabia and Yemen, an indication of the potential for spread and global health security importance of the virus.^2^ In Kenya, outbreaks are associated with heavy rainfall and flooding, which provides ideal conditions for mosquito vector multiplication and, consequently, disease emergence.^3,4^ As such, RVF epizootics are cyclic and periodic in nature, sometimes occurring as explosive outbreaks that cause significant morbidity and mortality in humans and animals.^5^ Mosquitoes of the *Aedes* species are considered to be the primary vectors, whereas the *Culex* and *Anopheles* species and other biting flies have been reported to be the main secondary, amplifying vectors that propagate transmission after emergence.^6,7^ Susceptible livestock, primarily sheep, goats, cattle, and camels, are infected through bites of infected mosquitos and through mechanical transmission by biting flies.^7^ On infection, the disease in animals is characterized by fever, abortion storms, and high mortality rate, especially among young livestock.^8,9^ Spillover of infection from animals to humans occurs through direct contact with infected animal fluids and tissues and consumption of contaminated animal products. Human infection through bites of infected mosquito, although rare, has been reported.^10^ Acute human infections present as a self-limiting febrile illness with nonspecific symptoms; however, a small proportion of cases proceed to a more severe disease that may present with ocular complications, encephalitis, hemorrhagic fever, jaundice, and other signs of liver malfunction.^11^

In Kenya, RVF is a priority zoonotic disease because of the high morbidity and mortality, frequency of outbreak events, and socioeconomic impacts during outbreak events.^12^ The last major outbreak in 2006–2007 resulted in approximately 340 human cases, 90 human deaths, and economic losses of more than US$32 million in direct livestock mortality and indirect losses, partly due to impediment to trade.^10,13^ As such, early detection and response to RVF outbreaks in animals before spillover to humans is a primary objective of the country’s animal health surveillance system.

In May 2018, the Kenya Meteorological Department reported that some regions of the North Eastern region of Kenya had received three times the expected annual rainfall in the period between March and May 2018.^14^ In the first week of May 2018, the Kenya Directorate of Veterinary Services (KDVS) activated a community-based, syndromic surveillance (SS) system to monitor occurrence of RVF-associated syndromes in livestock. This system had been previously used in 2015–2016 in 22 RVF high-risk counties in Kenya.^15^

On May 25, 2018, reports of mass abortions and mortality of young sheep, camels, and goats were reported to the KDVS by the animal health services in Eldas subcounty, Wajir County. By June 4, 2018, four human mortalities from the same subcounty, with a history of febrile and hemorrhagic illness, were reported to national human health services. On June 7, 2018, patient samples were submitted for laboratory testing at the Kenya Medical Research Institute (KEMRI), Nairobi. Two of three patients tested positive for RVF virus by reverse transcription–PCR (RT-PCR). A national outbreak of RVF was declared on June 7, 2018. And, consequently, a multidisciplinary field investigation team was deployed on June 13, 2018, to investigate the outbreak and assist with response and control efforts. Here, we report findings from the field investigation carried out among humans and livestock in Wajir, and subsequent outbreaks in humans in Marsabit and Siaya counties, Kenya.

## METHODS

### Study site.

**Figure 1.**
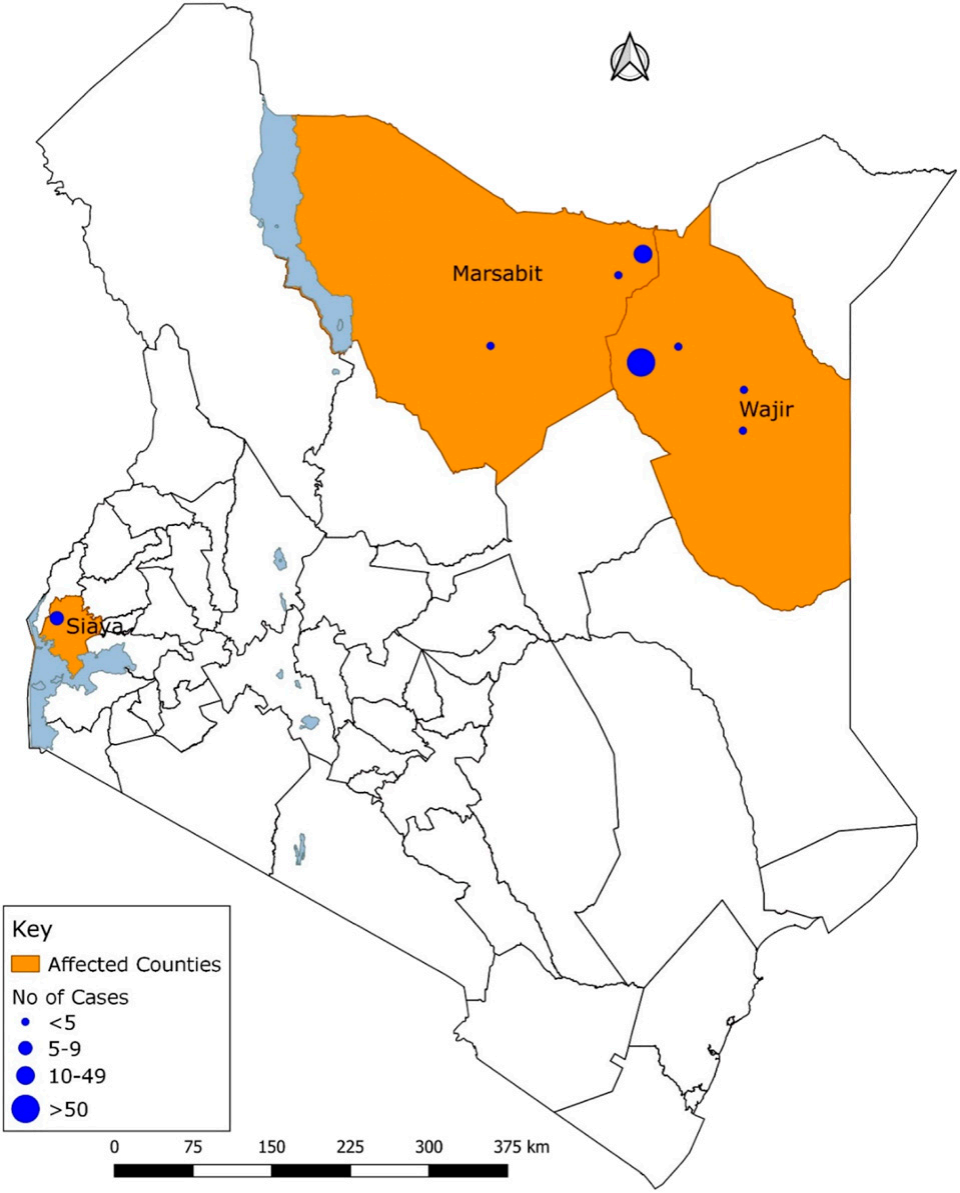
Spatial distribution of human Rift Valley fever cases (probable and confirmed) by counties, Kenya, May–June 2018 (*n* = 106). This figure appears in color at www.ajtmh.org.

The study was conducted in Wajir and Marsabit counties in northeastern Kenya and Siaya County in southwestern Kenya from May to June 2018 (Figure 1).

### Case detection and case ascertainment.

The investigation case definition was adapted from the WHO.^16^ A suspect case of RVF was any person presenting in health facilities in any of the affected counties with a fever (> 37.5°C) or a 2- to 6-day history of fever of unknown origin and sudden onset of flu-like symptoms such as muscle pain, joint pains, and headache with or without ocular disease, meningoencephalitis, or hemorrhagic fever. A probable case was a suspect case with close contact with sick or dead livestock (cattle, goats, sheep, and camels) at least 14 days before the onset of illness or a person who died with hemorrhagic-like signs between April and June 2018. Contact with livestock was defined as drinking of unpasteurized milk or any activity that results in exposure to animal blood and body tissues including and not limited to slaughtering, care of sick animals, veterinary procedures, disposal of products of abortions, and assisted animal birthing procedures.

A confirmed case was a suspect case with laboratory confirmation of the presence of anti-RVF virus IgM by ELISA or RVF RNA by RT-PCR.

We reviewed medical records for the 1-month period preceding the outbreak and conducted human active case finding using community-level key informants and door-to-door case ascertainment in affected areas in the three counties. Additional cases were identified through snowball sampling, that is, already identified suspected cases assisted with recruitment of other people with similar symptoms within the village. For livestock, the case definition was adapted from the World Organization for Animal Health (OIE) terrestrial manual.^17^ A suspect livestock case was any animal presenting or with a history of abortions or deaths of young animals (< 3 months old) or hemorrhagic syndrome in individual animals. A confirmed herd was a suspected herd with a laboratory confirmation of the presence in serum of anti-RVF virus IgM by ELISA in any individual animal within the herd. During the outbreak, livestock case finding was conducted in Wajir alone by tracing back human cases to their residence and sampling all animals the human cases had contact with 2 weeks preceding their illness. Additional livestock cases were traced through key informants who identified livestock herds presenting with RVF-like syndromes (abortions and deaths in young animals less than 3 months) in affected villages.

### Field data collection.

Structured questionnaires were administered to all human cases to collect data on demographics (age, gender, marital status, occupation, and education status), travel history, and clinical (illness history and outcome) and exposure information. Proxy interviews (close relatives) were conducted to obtain information on mortalities linked to the outbreak and minors (children younger than 10 years) or critically ill cases.

For livestock, a separate structured questionnaire was administered to owners to collect information about the herd characteristics (age and sex of animals) and clinical information (onset of illness, and presence of hemorrhagic symptoms, abortions, and mortalities).

### Collection and testing of human samples.

In each of the outbreak areas, 5 mL of blood was collected from suspected human cases, processed to serum, aliquoted into sterile bar-coded cryovials, and transported in liquid nitrogen dry shippers to the National Public Health laboratory and KEMRI viral hemorrhagic fever (VHF) laboratory for testing. Specimens were tested for the presence of Rift Valley Fever Virus (RVFV)-specific IgM antibodies using capture ELISA and for viral RNA using conventional RT-PCR as described using standard manufacturer operating procedures.

A laboratory-derived test provided by the Diagnostic Systems Division, United States Army Medical Research Institute of Infectious Diseases was used to detect the presence of IgM antibodies.^18^ Briefly, a commercial antihuman IgM antibody (goat antihuman IgM, Kirkegaard & Perry Laboratories, Gaithersburg, MD) was coated on 96-well Nunc immunoplates (Thermo Fisher Scientific, Roskilde, Denmark). Plates were then washed using a wash buffer (phosphate buffer saline [PBS], pH 7.4, 0.01 merthiolate, and 0.1 Tween-20) followed by addition of the RVF IgM positive control, negative control, and respective samples all diluted 1:100 in diluent buffer (PBS, pH 7.4, 0.01 merthiolate, 0.1 Tween-20, and 5% skim milk). Plates were incubated at 37°C for 1 hour, washed, and 100 μL of the RVF antigen (RVFV ZH 501 strain) solution added in one half of the test wells and a corresponding negative antigen (same dilution) added in the other half of the test wells. This was followed by a 1-hour incubation at 37°C. Plates were washed and 100 μL of RVF-specific detector antibodies (anti-RVF hyperimmune mouse ascitic fluid) added to each well and incubated for 1 hour at 37°C. Plates were washed and 100 μL of goat anti-mouse IgG, heavy and light chain specific conjugate (Kirkergard & Perry, catalog 074-1806) added in all the wells and incubated for 1 hour at 37°C. The plates were washed and 100 μL of the ABTS substrate (Kirkergard & Perry, Cat. No. N8 50-62-00) added followed by 30-minute incubation at 37°C. The optical density (OD) value was read with a spectrophotometer at 405 nm and the adjusted OD calculated by subtracting the OD of the negative/mock antigen–coated wells from the positive antigen–coated wells. The OD cutoff was calculated as the mean of the adjusted OD of the negative control sera plus three times the SDs. Any sample with OD ≥ 0.2 was considered positive.

### Molecular testing.

Assays were performed using real-time RT-PCR. RNA was extracted from the whole blood specimens using QIAamp Viral Mini Kit following the manufacturer’s protocol (QIAGEN GmbH, Hilden, Germany). Primers and probes targeting the G2 gene of the virus were then used to amplify a 94-nucleotide fragment using a TaqMan real-time RT-PCR assay. The PCR amplification of targeted viral sequence was performed in a 25-μL reaction mix containing 12.5 μL of 2× RT-PCR buffer,1 μL of 25× RT PCR enzyme, 0.25 μL of forward primer (40 µM), 0.25 μL of reverse primer (40 µM), 0.25 μL of probe (40 µM), and 5.75 μL of nuclease-free water. Five microliter of RNA template was then added to the reaction mix. An RVFV-positive control (RNA extracted from RVFV positive sample) and a negative control were included in the PCR reaction setup. Amplification was performed using Rotor-Gene^®^ Q real-time PCR system (QIAGEN GmbH), reverse transcription was performed at 50°C for 30 minutes followed by Taq activation at 95°C for 15 minutes, and then PCR was conducted for 45 cycles at 94°C for 15 seconds and 60°C for 1 minute. Following PCR analysis, any sample with a cycle threshold ≤ 37 was considered positive. The primers and probes used for the real-time PCR for this study are shown in the Annex.

### Collection and testing of animal samples.

Whole blood samples were collected by jugular venipuncture using Vacutainer EDTA collection tubes; the serum was processed by centrifugation, aliquoted, and kept in cooler boxes before transportation to the Central Veterinary Laboratory in Nairobi for testing.

Serum samples were tested for the presence of RVFV-specific IgM antibodies by capture ELISA using the IDvet diagnostic kit (ID Screen^®^ RVF IgM capture [ID-vet, Grabels, France]) per the manufacturer’s recommendations. Briefly, 10 µL of test and control serum samples were added to a microplate precoated with anti–bovine–ovine–caprine IgM antibody. The plate was incubated for 1 hour at 37°C and washed three times. Fifty microliters of RVF nucleoprotein was added, and the plate was incubated for 1 hour at 37°C. Fifty microlitres conjugate (anti-RVF virus nucleoprotein conjugated to horse radish peroxidase) was added to all wells and incubated at 37°C for 1 hour. After washing, 100 µL substrate solution was added to each well and incubated in the dark for 15 minutes at room temperature followed by adding 100 µL stop solution. The plate was read at 450 nm, and the OD was recorded. The test was valid if the net OD of the positive control was > 0.350, and the ratio of the mean values of the net positive and negative control ODs was > 3. Samples with percent positivity of ≥ 50% were considered positive, ≤ 40% negative, whereas results between 40% and 50% were equivocal and repeated.

### Data management and analysis.

Data from human cases and herd owners were entered, cleaned, and analyzed using Epi Info (CDC, Atlanta, GA) and Microsoft Excel (Microsoft Corp., Redmond, WA). We calculated means for continuous variables and proportions for categorical variables. Analysis was conducted only for laboratory-confirmed cases. Case fatality rates (CFRs) and attack rates (ARs) in humans by age-group, gender, and counties based on projections from national census data were calculated.

### Ethical considerations.

This investigation was conducted as part of an outbreak response to a public health emergency and did not require an approval from institutional ethics review committee; however, permission was obtained from national and county government authorities responsible for human and animal health. Verbal consent was obtained from all study participants aged ≥ 18 years; minors (aged < 18 years) were interviewed after consent was provided by a legal guardian or parent. Confidentiality of the participants was ensured by replacing their names with unique identifiers. Case patients were given feedback on the results of their laboratory investigation through the nearest health facilities.

## RESULTS

### Magnitude and characteristics of human cases.

Between May 11 and June 30, 2018, 106 human RVF cases (76 probable and 30 confirmed) were identified in three counties: 78% in Wajir (*n* = 83), 14% in Marsabit (*n* = 15), and 8% (*n* = 8) in Siaya. Of 80 suspected cases whose specimens were collected, 30 (38%) were confirmed for RVF, 23 (77%) positive by RT-PCR, 6 (20%) by IgM ELISA, and 1 (3%) by both tests. Among the 30 laboratory-confirmed cases, 70% (*n* = 21) were from Wajir County, 27% (*n* = 8) from Marsabit, and 3% (*n* = 1) from Siaya. The median age of the confirmed cases was 27.5 years (interquartile range = 20), and 18 (60%) were male. All confirmed cases presented with fever, 73% with headache, 70% with arthralgia, 33% with hemorrhagic symptoms, and 13% with jaundice (Table 1).

**Table 1:** Clinical characteristics and laboratory test results of the Rift Valley fever–confirmed human cases in Kenya, May–June 2018

Characteristic	Frequency	Percent
Clinical features (*n* = 30)		
Fever	30	100
Headache	22	73
Joint pains	21	70
Bleeding	10	33
Muscle pains	6	20
Vomiting	5	17
Jaundice	4	13
Confusion	3	10
Dizziness	1	3
Laboratory tests (*n* = 80)		
Positive (*n* = 30)	30	38
RT-PCR	23	77
ELISA	6	23
Both (RT-PCR and ELISA)	1	3
Negative	50	63

RT-PCR = reverse transcription–PCR.

**Figure 2.**
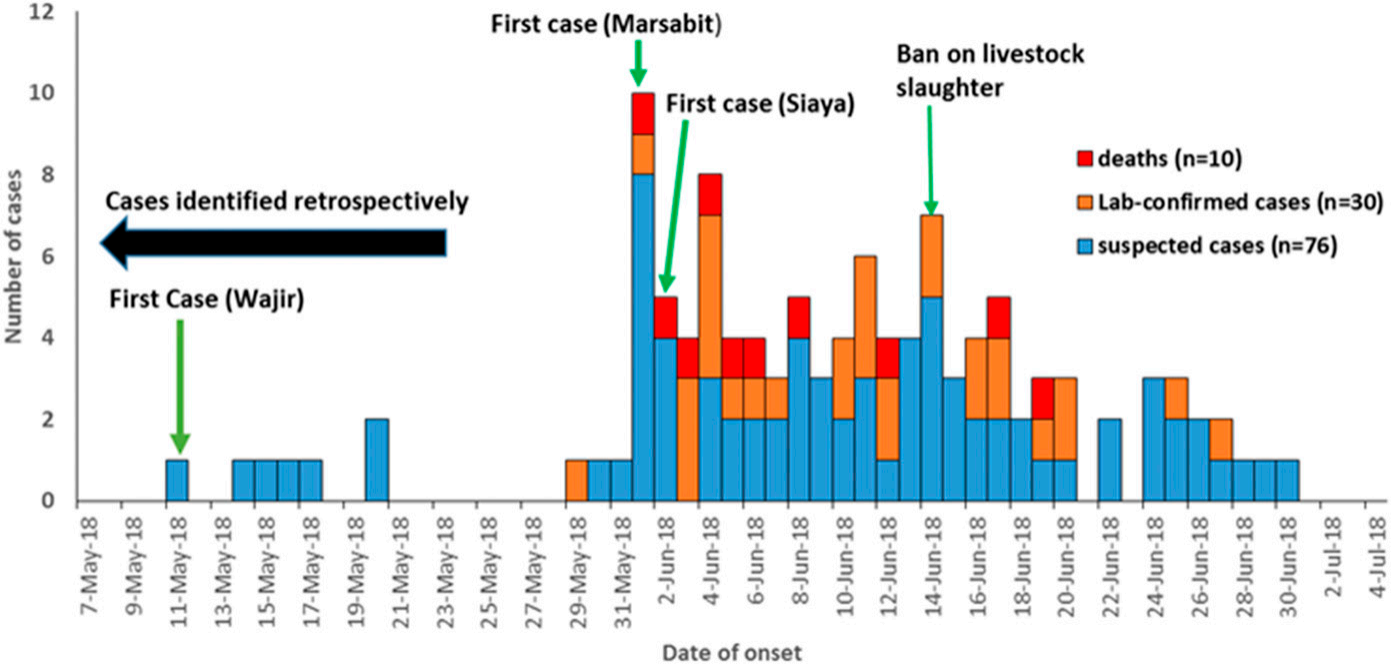
Epidemiologic curve of human Rift Valley fever cases by date of onset of symptoms, Kenya, May–June 2018 (*n* = 106). This figure appears in color at www.ajtmh.org.

We determined from medical records that the first suspected case was a 48-year-old herdsman from Eldas subcounty, Wajir County, who developed fever, headache, myalgia, dizziness, blurred vision, and bloody stool on May 11, 2018, 3 days after slaughter and consumption of meat from a sick camel. The case was admitted at Eldas subcounty hospital on May 16, 2018 and was put on supportive management and discharged on full recovery 3 days later. However, no laboratory investigation was carried out. There was no evidence of malaria infection by rapid diagnostic test assay or microscopy. Figure 2 describes the temporal distribution of human cases over the outbreak period.

The overall AR for the three counties was 1.4 per 100,000 persons, and the AR for Wajir County was 2.5 per 100,000 persons. Overall, the AR among males was 1.6 per 100,000 persons, and those aged ≥ 50 years reported an AR of 2.7 per 100,000 persons.

Of 30 confirmed cases, there were seven deaths (CFR = 23%): 6 (86%) males and 1 (14%) female. Males had a CFR of 33%, whereas age-group 0–14 years had a CFR of 67%. Marsabit County had the highest CFR of 38% (Table 2). Of 25 confirmed cases that were interviewed, 19 (76%) were herdsmen, 16 (64%) had no formal education, and 14 (56%) had visited health facilities to seek care, of which 9 (64%) were admitted (Table 3). More than half (64%, *n* = 16) of the confirmed cases reported consumption of meat from a sick animal, 28% (*n* = 7) drinking raw milk, and 28% (*n* = 7) preparation and cooking of meat.

**Table 2:** Distribution of Rift Valley fever ARs and CFRs in humans by county, age-group, and gender, Kenya, May–June 2018

Variable	Population (2017)	Cases	AR/100,000	Deaths	CFR (%)
Overall	2,190,284	30	1.4	7	23
Age-group (years)					
< 15	1,049,824	3	0.3	2	67
15–29	580,802	13	2.2	3	23
30–49	339,527	8	2.4	1	13
≥ 50	220,131	6	2.7	1	17
Gender					
Male	1,118,726	18	1.6	6	33
Female	1,071,558	12	1.1	1	8
County					
Wajir	852,963	21	2.5	3	14
Marsabit	372,931	8	2.1	3	38
Siaya	964,390	1	0.1	1	100*

AR = attack rate; CFR = case fatality rate.

* Siaya had only one confirmed case who died, hence the 100% CFR.

**Table 3:** Demographic and exposure characteristics of the Rift Valley fever confirmed human cases, Kenya, May–June 2018

Characteristic	Frequency (*n* = 25)	Percent
Visited health facility		
Yes	14	56
Admitted	9	64
Not admitted	11	36
No	11	44
Occupation		
Herdsman/woman	19	76
Unemployed	6	24
Level education		
No formal education	16	64
Primary incomplete	6	24
Secondary	3	12
Source of exposure		
Camels	15	60
Goat	12	48
Sheep	1	4
Type of exposure		
Consumed meat	16	64
Consumed raw milk	7	28
Handling and preparation of meat for cooking	7	28
Sheltered with herd	5	20
Handled aborted fetus	4	16
Handling carcass	4	16
Touch blood tissue	4	16
Assisted birthing process	3	12
Skinning	3	12
Slaughter	2	8
Treating sick animals	2	8
Contact with hides and skins	1	4

### Rift Valley fever outbreak in livestock in Wajir County.

**Figure 3.**
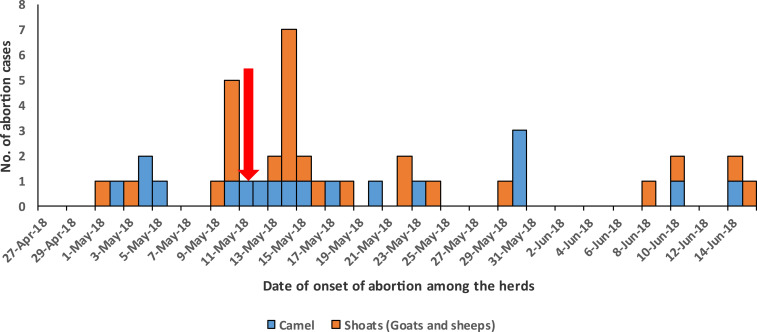
Temporal distribution of Rift Valley fever-suspected herds by date of onset of abortions among livestock, Wajir County, 2018. The arrow in red shows the suspected index case. This figure appears in color at www.ajtmh.org.

Key informants reported livestock abortions were first observed in Eldas subcounty, Wajir County, on May 1, 2018, reaching the peak on May 14, 2018. The suspected human index case was reported on May 11, 2018, a day after the first peak in livestock abortion cases on May 10, 2018 (Figure 3).

Interviews were conducted among 43 livestock owners whose herds met the suspected herd case definition. The total number of livestock in these herds was 5,963, consisting of 57% goats, 28% camels, and 14% sheep. Of 5,963 livestock in the suspect herds, 18% (*n* = 1,078) had abortions, 17% (*n* = 986) died, and 9% (*n* = 509) had a variety of clinical signs reported including fever, general malaise, and staggered gait (Table 4).

**Table 4:** Clinical characteristics of the RVF-suspected herds by animal type, Wajir County, Kenya, May–June 2018

Characteristic	Goats (*N* = 3,423), *n* (%)	Sheep (*N* = 851), *n* (%)	Camels (*N* = 1,689), *n* (%)	Total (*N* = 5,963), *n* (%)
Clinically sick animals	227 (7)	131 (15)	151 (9)	509 (9)
Deaths from suspected RVF	533 (16)	85 (10)	77 (5)	695 (12)
Death of young animals < 2 weeks	156 (5)	61 (7)	74 (4)	291 (5)
Abortions	747 (22)	176 (21)	155 (9)	1,078 (18)

RVF = Rift Valley fever.

During the investigation, 84 samples were collected from randomly selected livestock in the 43 suspected herds to confirm the presence or absence of RVF infection in livestock. Twenty-two (27%) of these sera samples were positive for anti-RVF IgM antibodies by IgM ELISA including 10/22 (46%) goats, 10/22 (46%) sheep, and 2/22 (9%) camels.

## DISCUSSION

This study describes the detection of RVF in humans in two high-risk counties of Kenya: Wajir and Marsabit, and a first time detection in Siaya County, an area previously perceived as low risk for RVF occurrence.^19–21^ Although this new detection is on a small geographical scale, it is worth noting that there is potential of spread of RVF at a national and regional scale. The occurrence in Siaya highlights the potential for spread and introduction into new ecological niches not previously classified as high risk.^20,22^ Once RVF virus is introduced into a new area, the virus can persist, and RVF outbreaks may be reported in subsequent years when climatic and environmental drivers are conducive for vector multiplication.^23,24^

Our investigation established a 30-day delay between the detection of suspected first case and the first laboratory-confirmed case. This is likely linked to low suspicion index for RVF cases among health workers coupled with limited capacity for RVF differential diagnosis with other acute febrile illnesses in the affected regions. The delayed detection of human cases could be associated with the low sensitivity of passive surveillance systems and the mild, nonspecific presentation of RVF, thus resulting to missed opportunities for early diagnosis.^8,11,25,26^ Like other previous RVF outbreaks in Kenya and elsewhere, we found a higher proportion of cases among males and livestock keepers.^27–29^ This is probably indicative of occupational risk and exposure to men who are primarily responsible for livestock herding, care of sick animals, and slaughter of livestock in pastoral communities; consequently, these men may be predisposed to close contact with blood and animal tissue from infected animals during outbreaks of RVF.^29,30^

The case fatality ratio in this outbreak (23%) was higher than that (16%) reported in previous outbreaks in Kenya but lower than that (46%) reported in Tanzania.^10,27^ This could be associated with poor health-seeking behavior, which resulted in few residents seeking clinical care and subsequent low diagnostic capacity.^31^

The outbreak was preceded by a period of prolonged heavy rainfall and resultant floodwater collections which creates conducive mosquitos-breeding habitats, the emerging potential for virus amplification and intensification of infections if the competent vectors are present.^32^

The first time that RVF was reported in Siaya may be due to autochthonous RVFV transmission by the presence of competent mosquito vectors. The epicenter of the outbreak in Siaya was suspected to be in villages around Lake Kanyaboli, a delta that drains into the expansive Yala Swamp. There is also high possibility of introduction of RVFV because of livestock trade and migration from other areas that may be at high risk of the disease. The lack of reported human and animal cases in the regions surrounding Siaya could be mainly attributed to weak surveillance characterized by low index of suspicion among healthcare workers (due to the traditional classification of these areas as low risk), misdiagnosis, and low reporting rates as opposed to the lack of active RVF transmission. This study had some limitations. About a quarter of the suspect human cases did not submit their specimens for testing because they were identified retrospectively at the community. This likely underestimated the number of confirmed cases reported during this outbreak. In addition, the CFR reported here is likely an overestimate and biased toward severe cases because it was based on confirmed cases, who were likely severe cases, and did not include mild cases identified at the community level that we did not have their specimen.

Most cases in this outbreak occurred among pastoralist livestock keepers with extensive animal contact. Public health education on risk reduction measures that target persons at high risk of exposure during outbreaks of RVF is critical to reduce infections. In addition, market closures and ban on slaughter of livestock at the community level may likely have significant impact in reducing human infections. The main interventions that were implemented as informed by the findings of the investigation included sensitization trainings with human and animal healthcare workers in outbreak areas on case detection and management, public outreach and surveillance, data sharing and infection, and prevention and control strategies. Overall, strengthening existing surveillance programs in all regions, irrespective of risk profile, for early detection of human and livestock cases, and rapid response is key to mitigate public health and socioeconomic impacts of RVF outbreaks. More specifically, strategies to improve disease reporting by pastoralists in high-risk areas through sensitization and equipping private and public frontline animal health workers with surveillance and reporting tools like risk assessment questionnaires and smartphones for electronic reporting were conducted. In under-resourced settings, innovative approaches such as the use of mobile phone–based SS to guide response have been identified as a useful complement to the traditional passive surveillance systems.^15^ In our investigation, we noted a significant proportion of suspect cases did not seek health care even though they fit the suspect case definition. This not only could be attributed to mild febrile illness on infection but also shows the need to educate vulnerable pastoral communities on RVF high-risk periods and the need to seek health care and report the presence of any signs of infection in their communities.

## Supplemental files

Supplemental materials
