# Supplementary material for: Epidemiological Investigation of a Rift Valley Fever Outbreak in Humans and Livestock in Kenya, 2018
Source: Am J Trop Med Hyg. 2020 Aug 3;103(4):1649–55. doi: 10.4269/ajtmh.20-0387 (PMC7543801; doi:10.4269/ajtmh.20-0387)
Supplement: Supplementary file 1 [file tpmd200387.SD1.pdf]

Appendix 1: Rift Valley Fever Investigation Questionnaire

June 2018, Wajir County, Kenya – Individual questionnaire

Section 1: Identifiers

Interview Date __ __ / __ __ / __ __ __ __ (dd/mm/year)

Interviewer Name/Code __ __

Household (HH) ID__ __ - ___ ___ ___ *(the first two digits are code for the village/center, then houses are numbered sequentially- For example the first house in Eldas should be given ID number EL-001. etc)*

No.of people in the HH_______

Participant ID __ __ - __ __ __ -- __ __ *(first 5 digits are household ID, followed by individual's ID in household. For example, the third individual enrolled from the first house in Eldas will be EL-001-03, etc)*

(a.) Person being interviewed *(circle)*:

1. Self	☐	4. Child	☐
2. Parent	☐	5. Sibling	☐
3. Spouse	☐	6. Relative	☐

(b.) Reason for proxy interview?

1. Person is a child ☐
2. Person has died ☐
3. Person is not at home ☐
4. Very ill ☐

Section 2: Personal Information

Name______________________________________

Sex

1. Female ☐
2. Male ☐

Age (in Years) _________

Marital status

1. Single ☐
2. Married ☐
3. Divorced ☐
4. Widowed ☐

Religion

1. Muslim ☐
2. Christian ☐
3. Traditional ☐

Occupation

1. Formal Employment ☐
2. Herdsman/Woman ☐
3. Housewife ☐
4. Farmer ☐
5. Student ☐

Level of education

1. Tertiary ☐

2. Secondary
3. Primary completed
4. Primary incomplete
5. No formal education
6. Madrassa/duksi

(i).What is your residence?

(a.) Sub-County ____________________
(b.) Ward ___________________
(c.) Village ___________________
d) Household Geocode : Lat : _____ Long : _____

(ii).Between the 1st May and Today have you traveled out of your village?

1. Yes ☐ 2. No ☐

a) If yes where?

Village		Ward	Sub-County	County
1.				
2.				
3.				

(iii).Number of days out of village between 1st May and today? _______

Section 3: History of Illness

1. Have you fallen ill between the period of 1st May and Today? (Yes/No**)**
2. If yes above, Did you have any of the following symptoms between 1st May and Today? If yes specify in the table below

**Symptom**	**Yes**	**No**		**Symptom**	**Yes**	**No**
Hotness of the Body				Bloody Stool		
Headache				Bloody Vomiting		
Muscle Aches				Cough with blood		
Joint Pains				Bloody vaginal discharge		
Malaise				Nose bleeding		
Vomiting				Bleeding gums		
Diarrhea				Skin purpura		
Chills				Blurred vision		
Abdominal pain				Confusion		
Dizziness				Convulsion		
Miscarriage /abortion				Coma		

Jaundice				Double vision		
				Blurred vision		

(i). Date of onset of illness (Fever if present) (dd/mm/year)……./…../……

(ii). How many days did the illness last? _______ Days

(iii). Outcome status:

1. Alive ☐
2. Dead ☐

iv) If Alive is the person

1. Admitted
2. Discharged
3. In community

(v).Did you seek medical care for the illness?

1. Yes ☐ 2. No ☐

(vi).Where did you seek for medical care? (Tick all that apply)

1. Dispensary	☐	2. Health center	☐	3. Hospital	☐
4. Traditional healers	☐	5. Religious healer	☐	6. Others specify………………….)	☐

(vii).Were you admitted to the hospital/health center (sleep overnight) for this illness?

1. Yes ☐ 2. No ☐

(viii). How long were you hospitalized?__________(in days)

(ix).What day did you first seek treatment (dd/mm/year)……./…../……

(x).Were you tested for malaria? Yes/No
(xi) If yes, what were the results? 1-Positive 2-Negative 3-Don't know

Section 4: Exposure Details: Period of Interest (between the 1st May and Date of interview)

(i). Did you consume or handle any meat, milk, blood from any **sick** animal between 1st May and today (date of interview)?

1. Yes ☐ 2. No ☐

(ii). If Yes which **sick** animal (Tick)?

1. Sheep	☐	2. Goat	☐	3.Cows	☐
4. Camels	☐	5. Donkeys	☐	6. Others, specify	☐ ___________________

(iii)Specifically concerning the SICK/dead animal, did you do any of the following?

		**Sheep**	**Goats**	**Camel**	**Cow**
	**Yes/**	**How many**	**How many**	**How many**	**How many**

	No	times	times	times	times
**Slaughter**					
**Skinning**					
**Herding (# days)**					
**Sleeping with herd (# nights)**					
**Milking**					
**Touch blood or tissue**					
**Assisted animals during birth/abortion**					
**Touch/handled aborted fetus/still births**					
**Handle animal dung**					
**Consumed meat**					
**Involved in preparing meat**					
**Consumed raw milk**					
**Contact with hides and skins**					
**Treating sick animal**					

(iv). If you were not involved in these activities, were you within 5 meters during any of these activities on SICK animals?

1. Yes ☐ 2. No ☐ 3. Don't Know ☐

(v). Between the 1st May and today, how many nights did you sleep outdoors? _____

(vi). How many nights did you sleep in the bush? _________

(ix). How much of the day did you spend outside between 1st May to date (not including sleeping)?

1. All day (dawn to dusk) ☐ 2. Most of day ☐ 3. Few hours ☐

(x). Do you have a bednet? 1. Yes ☐ 2. No ☐

(xii). When was the ITN last treated?

1. Never treated ☐ 2. Within 1- 3 months ago ☐
3. 3 – 12 months ago ☐ 4. Over 12 months ago ☐

(xiii). Between 1st May and today, did you sleep under bednet?

1. Every Night ☐ 2. Some Nights ☐ 3. Never ☐

(xiv). Did you use any mosquito repellants in between 1st May and today?

1. Yes ☐ 2. No ☐

(xviii). Did you spray or anyone else (MOH staff) spray (with insecticides) your house

between 1st May and today?

1. Yes ☐ 2. No ☐

(xix) Between 1st May and today, how many mosquitoes do you remember seeing outside/inside your house?

1. A lot/More than normal ☐ 2. Some/Normal amount ☐ 3. Few/Less than normal

(xx) Between 1st May and today, are there other people in this household who have become sick after consumption or contact with sick/dead animal meat, milk and blood? Yes/No
(xxi)If yes, how many?------------------

Section 5: RVF Knowledge Assessment

(xix).Have you ever heard of Rift Valley Fever before the current outbreak?

1. Yes ☐ 2. No ☐

(xx) If Yes, from whom?
a) Health care workers (b) radio (c) Television (d) community leaders (e) Barazas (f) family (g) neighbors/friends (h) schools
(xxi) Do you know what causes RVF? Yes/No
(xxii) If yes, how does one get RVF? (mark all that apply)
(a) Mosquito bites (b) Eating undercooked meat from a sick animal (c) drinking raw milk from a sick animal (d) slaughtering/skinning sick animals (e) handling abortus (f) assisting animal delivery of a sick animal (g) milking sick animals (h) contact with blood of a sick animal
(xxiii) Do you know the signs and symptoms of RVF virus disease in humans?

1. Yes ☐ 2. No ☐

(xxiv) If yes, what are the signs and symptoms? (mark all that apply)

Symptom		Symptom	
Hotness of the Body		Bloody Stool	
Headache		Bloody Vomiting	
Muscle Aches		Cough with blood	
Joint Pains		Bloody vaginal discharge	
Malaise		Nose bleeding	
Vomiting		Bleeding gums	
Diarrhea		Skin purpura	
Chills		Blurred vision	
Abdominal pain		Confusion	
Dizziness		Convulsion	
Miscarriage /abortion		Coma	
Jaundice		Double vision	
		Blurred vision	

(xxv) Do you think RVF is preventable? (Yes/No)
(xxvi) If Yes, How is it preventable? (Mark all that apply)

Vaccination of animals	
Use of mosquito Nets	
Avoid consuming uninspected meat/raw milk	
Use protective equipment when handling aborted fetus	
Avoid contact with fluids from sick animals	
Drain stagnant waters/clearing bushes	

END
