# Supplementary material for: Epidemiological Investigation of a Rift Valley Fever Outbreak in Humans and Livestock in Kenya, 2018
Source: Am J Trop Med Hyg. 2020 Aug 3;103(4):1649–55. doi: 10.4269/ajtmh.20-0387 (PMC7543801; doi:10.4269/ajtmh.20-0387)
Supplement: Supplementary file 2 [file tpmd200387.SD2.pdf]

**Appendix 2. RIFT VALLEY FEVER OUTBREAK ANIMAL INVESTIGATION FORM**

**A. Identifying Information**

County: ……… Sub-county: ………………..

Ward/Town/Village: …………….. Date of interview: …………….

Name of the respondent: (option: Herd owner, relative)

Contact of the respondent:

Village of residence:

HH number………Geocode…………….

**B. Herd information**

- Herd No.: ………. Species: (Sheep, Cattle, Camel, goat)
- Total No of Animal…
- No. of male:
- No. of Female:
- No. Pregnant:
- No. of sick animal:
- No. that aborted since 1st May 2018
- No. died since 1st May 2018
- No. slaughtered following sickness since 1st May 2018

**C. Clinical data for sick animal (two weeks and older)**

1. Date of onset of illness: (Date)

2. What Clinical signs did you observe in sick animal

    a) Animal not feeding
    b) dark smelly diarrhea or with blood
    c) Abortion
    d) blood in nasal discharges
    e) Swollen lymph nodes
    f) Jaundice
    g) Salivation
    h) General weakness
    i) Abdominal pain/colic
    j) Agalactia
    k) Recumbency
    l) Anorexia
    m) Still births

**D. Data on young animal**

3. Was there death in young Animal (< 2 weeks old)? Y/N

4. If yes how many died in your herd (number)

5. How long after onset did the illness last

    a) Less than 24 hours
    b) 24-48 hours
    c) more than 48 hours

6. What are the clinical signs/symptoms seen in young animal (check box)

    a) Fever
    b) Depression
    c) Too weak to suckle
    d) Sudden collapse and death
    e) Other signs and symptoms

**E. Data on cases of abortion**

7. When did you observe the first cases of abortion in the herd (Date)

8. How old in months was the pregnancy during the abortions?

9. Did you assist the animals during abortions?

10. If yes, did you use any protective gear, eg gloves?

11. How did you handle the dead fetuses? (option)

    a) Allow it to rot in the compound
    b) buried it
    c) Burnt it
    d) Threw it for vultures/scavengers

**F. Data on post- slaughter observations**

12. Did you slaughter any animals or sick ones between May 1st, 2018? Yes/No

13. What did you observe on the following organs after slaughtering the animal (Check box

    a) Meat/fascia – yellow tinged, dark red
    b) Liver – Enlarged, looking cooked, bronze to yellow color
    c) Under the skin/hide – blood spots
    d) Intestine – bloody content
    e) lymph nodes – swollen
    f) Lung – swollen

**G. General knowledge information** (herd owners/respondents)

14. Have you heard of rift valley fever? Y/N

15. What do you think transmits RVF in animal?

    a) Mosquito
    b) Ticks
    c) Biting flies
    d) Don't Know
16. Do you know that it can be transmitted from animal to human? Y/N

17. If yes, what can you do to stop getting it from animal (Check box)

    a) Don't eat meat from sick animal
    b) Don't slaughter/skin sick animal suspected to have died of RVF
    c) Don't touch aborted fetus
    d) Don't use/drink milk from sick animal
    e) Avoid contact with body fluids from animals suspected to have RVF Disease
    f) Don't know
18. Do you know that it can be prevented in Animal? Y/N
19. If Yes, what methods?
    a) Vaccination
    b) Treatment
    c) Avoiding contact with sick herd
    d) Quarantine
20. Have you ever vaccinated your animals against RVF? Y/N

21. If yes, when was the last vaccination done?

    a) Less than 6 months ago
    b) 6 months-1 year
    c) 1year- 2 years
    d) More than 2 years

22. How do you manage sick animal

    a) Treat it myself
    b) Request veterinary officer to treat
    c) Request Community animal health worker to treat
    d) Slaughter for consumption
    e) Allow it recover on it's own
23. How do you manage animal carcass?

    a) Bury
    b) Burn
    c) Leave it to rot
    d) Skinning
    e) Feed to scavengers

**H. Laboratory data**

24. Animal species: Cattle, Goats, Sheep, Camel)
25. -Date of sample collection:
26. Sample Type:  Serum, Blood, Tissue
27. Number of samples:
28. Type of test: ELISA, PCR
29. Name of the Lab.: CVL, ILRI
30. Date Lab received:
31. Date of result:
32. Status: Positive / Negative

**I. Final classification of the herd**

33. Lab. Confirmation: Lab (confirm test used)
34. Suspected: Clinical
35. Date of diagnosis:

Approve by ………………. Field investigator.

**END**
